# Trauma-related acute kidney injury during inpatient care of femoral fractures increases the risk of mortality: A claims data analysis

**DOI:** 10.1016/j.ajmo.2022.100009

**Published:** 2022-02-26

**Authors:** Gisela Büchele, Martin Rehm, Rebecca Halbgebauer, Dietrich Rothenbacher, Markus Huber-Lang

**Affiliations:** a Institute of Epidemiology and Medical Biometry, Ulm University, Ulm 89081, Germany; b Institute of Clinical and Experimental Trauma-Immunology (ITI), University of Ulm, Germany

**Keywords:** Trauma, Acute kidney injury, Mortality risk, Epidemiology, Outcome

## Abstract

*Objectives:* Although femoral fractures can hit anyone, they carry an especially high burden in the elderly and are multifaceted in their injury pattern, related complications, and subsequent therapeutic strategies. An often underestimated posttraumatic risk is the development of trauma-related acute kidney injury (TRAKI). However, for TRAKI, no outcome study with a large data approach exists addressing fractures. Therefore, we analyzed the development of TRAKI in regard to different covariates and quantified the association of TRAKI with overall mortality.

*Design:* Retrospective cohort study with claims data.

*Setting and participants:* 119,000 patients from Germany with femur fracture.

*Methods:* We calculated cumulative mortality, mortality rates per 100 person-years (both occurring within 180 days after fracture), and adjusted hazard ratios with 95%-confidence intervals (CI).

*Results:* Patients with femur shaft fractures showed an incidence of 6.1% for TRAKI, followed by patients with femur neck fractures with an incidence of 5.7%, and by patients with distal fractures with an incidence of 4.5%, respectively. Overall, in patients with any femur fracture, we found a 3.17-times higher mortality rate (95%-CI: 3.02-3.26) during 180 days of observation in patients who developed TRAKI in comparison to patients without. The risk for development of TRAKI was significantly increased with increasing TRAKI stage, age, and time until surgical intervention.

*Conclusions and implications:* In conclusion, patients suffering from proximal-, shaft-, and distal femoral fracture face an over 3-times higher 180 day-mortality rate in case of posttraumatic TRAKI, which should be considered in peri-traumatic care to improve the long-term outcome of these patients.

## Highlights

• > 5% of patients with femur fracture developed a trauma-related acute kidney injury.
• Patients with trauma-related acute kidney injury had a 3.2-higher mortality rate.
• Highest mortality for kidney injury was in patients with distal femoral fracture.
• Fracture-patients with trauma-related acute kidney injury need special attention.

## Introduction

Femoral fractures are the most common fracture treated in German hospitals.^1^ It can hit anyone along the whole life span, but especially the elderly are at high risk. These fractures are multifaceted in their injury pattern, therapeutic consequences, related complications, and outcome. Globally, increasing life expectancy causes increasing rates of femur fractures.^2^ Accordingly, in a German registry study, the numbers of any kind of femoral fracture increased in the elderly over the last 15 years, particularly in females, whereas they decreased slightly in younger patients.^3^ Anatomically, femur fractures are differentiated as proximal (femoral neck, per-, inter-, and sub-trochanteric), diaphyseal (femoral shaft), and distal (supra and per-/inter-condylar). Whereas proximal femoral fractures are often caused by low-energy trauma (e.g. ground-level fall) and often hit elderly persons, diaphyseal fractures occur predominantly in younger persons hit by a high-energy trauma vector (e.g. high-velocity motorbike crash). Differential consequences of femur fractures also include the degree of blood loss: whereas persons with femoral neck fracture rarely face any significant blood loss, femoral shaft fracture can result in life-threatening hemorrhagic shock.^4^ In industrial countries, femoral fractures are mainly treated by timely surgical osteosyntheses (including intramedullary nailing, plates, screws, external fixator).

Concerning post-fracture consequences, in the last century, mortality after femoral fracture, especially in the elderly, was high. It was caused by immobility-induced complications including pneumonia, bedsore, urogenital infection,^5^ but also on hemorrhagic-shock-related organ failure, the latter threatened the younger patients too. In the case of hip fracture in the elderly, surgical advancement and modern ortho-geriatric co-management could improve the outcome.^6^ However, long-term results after femur fractures cause a considerable burden and remain challenging for the individual patient and the society.^7^ Besides the fracture itself other risk factors for survival^3^ such as age, sex, and comorbidities are known and especially the development of acute kidney injury (AKI),^8^ which is often underestimated, may influence the outcome

The induction and progression of trauma-related AKI (TRAKI) involve a complex immuno-pathophysiology as recently reviewed in detail^9^. Clinically, besides patient-based risk factors (e.g. comorbidities, advanced age, fragility) also trauma-specific risk factors (e.g. hemorrhagic shock, traumatic brain injury, sepsis) favor the development of TRAKI.^9^^,^^10^ Focusing on femoral fractures beside preexisting comorbidities chronic kidney failure is one driver of TRAKI in the case of intertrochanteric and neck fractures.^11^, ^12^, ^13^, ^14^ The world hip trauma evaluation study has recently shown in more than 8,600 patients that the risk of TRAKI was 1.3% during the first 4 months after hip fracture.^15^ Several further primary studies with smaller cohorts exist, mainly evaluating hip fracture surgery (e.g. femoral neck) in the elderly which univocally stated a poorer outcome in the case of early postoperative AKI,^16^^,^^17^ even in a long-term follow up.^18^^,^^19^

Concerning the clinical diagnosis of AKI as an abrupt decline in renal function, various criteria have been defined such as the RIFLE criteria in 2004, which provide a multidimensional staged definition^20^ and a modification by the AKI Network (AKIN) criteria.^21^ In 2012, the Kidney Disease Improving Global Outcomes (KDIGO) classification system has been introduced harmonizing the RIFLE and AKIN criteria.^22^ To reflect these differentiations of AKI severity, a staged coding system for AKI has also been introduced in health claim companies in 2015, which may result in different incidence and outcome assessments when investigating historical groups.

However, in the context of TRAKI, although there is a great need for deeper insights, no study with a large data approach exists addressing fractures along the whole femur and along the whole life span and the outcome. Therefore, in the present study, we performed a large claims data analysis of more than 119,000 patients with the diagnosis of femur fracture and analyzed the development of TRAKI in regard to the different fracture regions, age, sex, comorbidities, and care need. In addition, we quantified the association of TRAKI with overall mortality within 180 days after hospital admission taking potential covariates into consideration.

## Material and methods

### Data source and study population

**Fig. 1:**
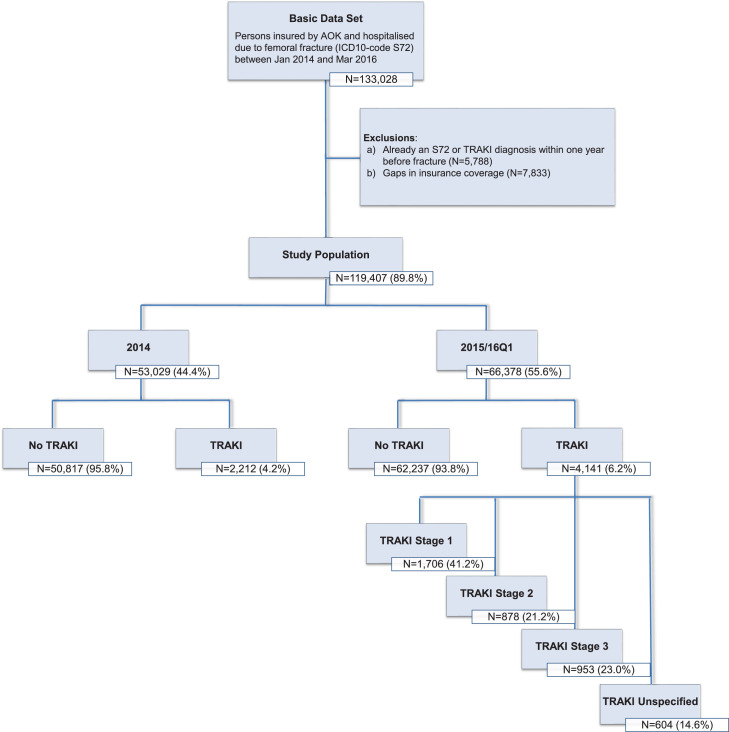
Study Population N=number of participants; TRAKI=trauma-related acute kidney injury; AOK=Allgemeine Ortskrankenkasse (a German health claim company); ICD-10 = International Classification of Disease (10th revision); Q1 = 1st quarter

The basic dataset consisted of 131,028 patients admitted to hospitals with a femoral fracture (ICD-10: S72) between 01.01.2014 and 31.03.2016 in Germany and insured by the *Allgemeine Ortskrankenkasse* (AOK). AOK is Germany's largest health claim company and covers nearly one-third of Germany's population of 82.5 million from cradle to grave. Patient-related health insurance claims data were provided by the scientific institute of the AOK (*Wissenschaftliches Institut der AOK*, WIdO). Exclusions of patients were due to former diagnoses of femoral fracture or TRAKI one year before the admission date (*n* = 5788) or due to gaps in the insurance history (*n =* 7833). The study population is presented in the flow chart in Fig. 1. As this study comprised analysis of anonymized routine data, specific ethical approval was not considered necessary by the ethics committee of Ulm University.

### Dependent and independent variables

The outcome was mortality within 180 days after hospital admission. Patients with an ICD-10 coding of N17 during their hospital stay were identified to have a TRAKI (Fig. 1). Since 2015 a detailed stage coding regarding the severity of the AKI was newly introduced, three gradually increasing stages and “not specified” were additionally available in the routine data.

### Covariates

Age in years at the index fracture and sex were documented in the claims database. Information about care need (yes/no) and nursing home status was available on a quarterly basis and was linked to admission data. For adjustment, information from the quarterly period before the fracture was used (Q-1). An assessment of the degree of ‘care need’ of frail care recipients is compulsory under the German Social Security Code XI (‘*Sozialgesetzbuch’*). In order to claim for long-term care benefit, people must need daily a minimum of 90 min of assistance with basic activities of daily living such as washing, eating, or dressing and of instrumental activities of daily living such as cleaning or shopping. Assessment of care need is performed by the medical service of the health insurance funds. Being categorised in one of the three levels of care according to the extent of care required can, therefore, be used as a surrogate marker of disability.^23^ Time to surgery has been shown to influence mortality.^24^ Therefore, time from hospital admission to surgery was determined and used as a covariate. A medication-based co-morbidity score was applied to account for multi-morbidity.^25^ To control for confounding all covariates were included in the regression models.

### Statistical analysis

Baseline characteristics were described by means and standard deviations or absolute numbers and percentages. Cumulative mortality (in %) and mortality rates per 100 person-years were calculated for deaths occurring within 180 days after fracture.

Cox proportional hazards regression models were used to estimating hazard ratios for mortality with 95% confidence intervals (using the coxph function in the R package survival). All models were adjusted for established covariates such as age, sex, need for care, nursing home status, days from hospital admission to surgery, and medication-based comorbidity score. Age and the comorbidity score were continuous, all others were categorical variables. The proportional hazard assumption was checked by inspection of Schoenfeld-residuals and found to be fulfilled for all models. The model fit parameter *concordance* ranged between 0.72 and 0.77 indicating good model fits. Statistical significance was used in an exploratory manner. The analysis was performed with R version 4.0.2 (R Foundation for Statistical Computing, Vienna, Austria).

## Results

Within the total study population of *N* = 119,407 patients being hospitalized due to femoral fracture (S72), *N* = 6,353 patients developed a trauma-related acute kidney injury (TRAKI). (Fig. 1) After the introduction of the AKI stages as coding imperative, there was a distribution of 41% of patients in stage 1, 21% resp. 23% in stages 2 and 3, and 15% of patients were unspecified.

The share of patients with TRAKI increased from 2014 with 4.2% (4.0–4.4%) to 2015/16Q1 with 6.2% (6.1–6.4%), which shows a statistically significant increase. (Supplement Table A) No further relevant differences were seen between the analysed years Table 1. displays the baseline characteristics by trauma-related TRAKI diagnosis. Overall, patients had a mean age of 80.7 years and 6.2% were between 80–89 years old. In addition, 73.2% were female.

**Table 1:** Baseline characteristics by trauma-related acute kidney injury (TRAKI) diagnosis.

	No TRAKI	TRAKI	Overall
Characteristic	(*N* = 113054)	(*N =* 6353)	(*N =* 119407)
Year 2015/16Q1, N (%)	62237 (55.1)	4141 (65.2)	66378 (55.6)
Age (years)	80.5 (12.5)	84.0 (8.2)	80.7 (12.3)
Age group, N (%)			
<60	6295 (5.6)	97 (1.5)	6392 (5.4)
60-69	7471 (6.6)	240 (3.8)	7711 (6.5)
70-79	25878 (22.9)	1144 (18.0)	27022 (22.6)
80-89	51889 (45.9)	3285 (51.7)	55174 (46.2)
≥90	21521 (19.0)	1587 (25.0)	23108 (19.4)
Female, N (%)	83118 (73.5)	4272 (67.2)	87390 (73.2)
Care need, N (%)	58160 (51.4)	3819 (60.1)	61979 (51.9)
Nursing home, N (%)	21898 (19.4)	1143 (18.0)	23041 (19.3)
Comorbidity score	3.9 (2.1)	4.4 (2.1)	4.0 (2.1)
Fracture diagnosis ICD-10, N (%)			
S72.0-2*	102305 (90.5)	5858 (92.2)	108163 (90.6)
S72.3*	8328 (7.4)	511 (8.0)	8839 (7.4)
S72.4*	5626 (5.0)	252 (4.0)	5878 (4.9)
S72.7-9*	3066 (2.7)	196 (3.1)	3262 (2.7)
Days until surgery, N (%)			
0	39973 (35.4)	1987 (31.3)	41960 (35.1)
1	44867 (39.7)	2444 (38.5)	47311 (39.6)
2	11237 (9.9)	775 (12.2)	12012 (10.1)
≥3	9601 (8.5)	840 (13.2)	10441 (8.7)
NA	7376 (6.5)	307 (4.8)	7683 (6.4)
Hospital stay (days)	18.9 (12.6)	26.1 (21.9)	19.3 (13.4)

Values are reported as n (%) or mean (SD).

N=number of participants; ICD-10 = International Classification of Disease (10th revision); Q1 = 1st quarter; * S72.0-2: Femoral neck fracture, pertrochanteric or subtrochanteric fracture, S72.3: Fracture of the femoral shaft, S72.4: Distal fracture of the femur, S72.7-9: Multiple fractures of the femur, fractures of other parts of the femur, or fracture of the femur, part unspecified.

There were further differences between patients with and without TRAKI in age, sex, care need, and co-morbidity indicating an older and more vulnerable patient group developing TRAKI. Patients with TRAKI had a longer time in the hospital before surgical intervention. Furthermore, the duration of the hospital stay was prolonged for about 7 days.

**Fig. 2:**
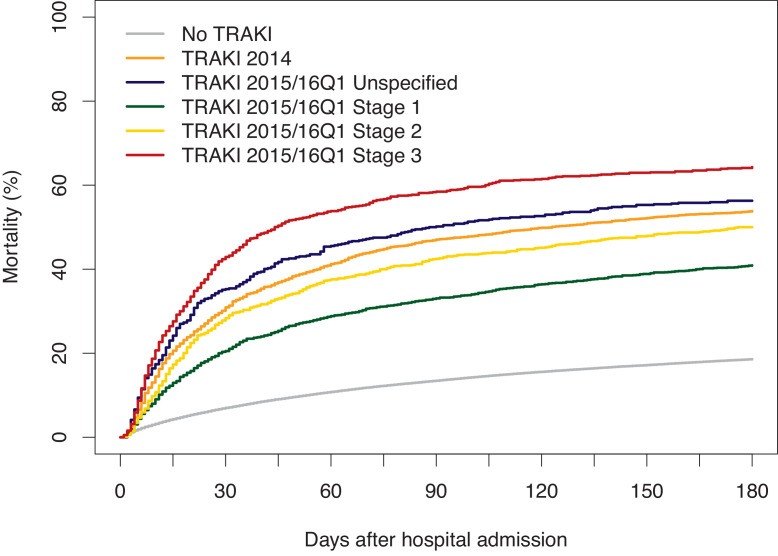
Mortality compared between patients hospitalized due to femoral fracture without and with TRAKI in general (2014) and different stages (2015/16Q1) TRAKI=trauma-related acute kidney injury; Q1 = 1st quarter.

In total, more than half of all patients with TRAKI died within 180 days after hospital admission. Compared to patients without TRAKI, patients with TRAKI had a 3.2-time increased mortality risk. (Table 2) In 2014 without stage differentiation, the mortality risk was 3.5-times higher. In 2015/16Q1 we can see a dose-effect relationship with increasing risks for increasing stages. The group with unspecified TRAKI showed a relatively high risk. In 2014, there seems to be a balanced mortality risk compared to a weighted mean risk of the different stages in 2015/16Q1 (Fig. 2).

**Table 2:** Cox regression analysis for mortality within 180 days after femoral fracture by year of fracture (*n* = 119,407, deaths = 24,297 (20.3%)).

Fracture Year	TRAKI Diagnosis/ Stage	Subjects at Risk, N	Subjects with Event, N (%)	Rate per 100 person-years	Hazard Ratio (95% CI)*
Overall	No TRAKI	113,054	21,016 (18.6)	43.0	Ref.
	TRAKI	6,353	3,281 (51.6)	174.9	3.17 (3.06-3.29)
2014	No TRAKI	50,817	9,228 (18.2)	41.8	Ref.
	TRAKI Unspecified	2,212	1,191 (53.8)	187.9	3.50 (3.29-3.72)
2015/16Q1	No TRAKI	62,237	11,788 (18.9)	44.0	Ref.
	TRAKI Stage 1	1,706	698 (40.9)	118.6	2.16 (2.00-2.33)
	TRAKI Stage 2	878	439 (50.0)	163.5	2.92 (2.66-3.22)
	TRAKI Stage 3	953	613 (64.3)	276.0	4.81 (4.43-5.22)
	TRAKI Unspecified	604	340 (56.3)	208.5	3.58 (3.21-3.99)

*Adjusted for age, sex, care need, nursing home, comorbidity score, and days until surgery.

TRAKI=trauma-related acute kidney injury; CI=confidence interval; ICD-10=International Classification of Disease (10th revision); N=number of participants; Q1 = 1st quarter.

Regarding different kinds of S72, the highest risk for mortality of TRAKI-patients was in patients with distal femoral fracture (S72.4) (adj. HR = 3.7 (95% CI: 3.1–4.5); Table 3). However, according to the confidence intervals, there was no statistically significant difference compared to the risk of other S72 fracture types.

**Table 3:** Cox regression analysis for mortality within 180 days after femoral fracture (S72) by ICD-10 diagnosis/category (*n* = 119,407, deaths = 24,297 (20.3%)).

ICD Diagnosis/Category	TRAKI Diagnosis	Subjects at Risk, n	Subjects with Event, n (%)	Rate per 100 person-years	Hazard Ratio (95% CI)*
S72.0-2*	No TRAKI	102305	19474 (19.0)	44.2	Ref.
	TRAKI	5858	3039 (51.9)	176.0	3.14 (3.02–3.26)
S72.3*	No TRAKI	8328	1268 (15.2)	34.3	Ref.
	TRAKI	511	234 (45.8)	143.9	3.20 (2.78–3.68)
S72.4*	No TRAKI	5626	858 (15.3)	34.3	Ref.
	TRAKI	252	126 (50.0)	164.2	3.72 (3.08–4.50)
S72.7-9*	No TRAKI	3066	520 (17.0)	38.6	Ref.
	TRAKI	196	97 (49.5)	158.3	3.18 (2.55–3.98)

*Adjusted for age, sex, care need, nursing home, comorbidity score, and days until surgery.

TRAKI=trauma-related acute kidney injury; CI=confidence interval; ICD-10 = International Classification of Disease (10th revision); * S72.0-2: Femoral neck fracture, pertrochanteric or subtrochanteric fracture, S72.3: Fracture of the femoral shaft, S72.4: Distal fracture of the femur, S72.7-9: Multiple fractures of the femur, fractures of other parts of the femur, or fracture of the femur, part unspecified.

## Discussion

In the present retrospective cohort study from Germany, we investigated a nationwide large claim data set of more than 119,000 hospitalized patients, most of them elderly, with a femur fracture including the proximal, diaphyseal, and distal part, and found overall in more than 5% development of TRAKI. Of note, these patients lacked any reported signs of kidney injury before the trauma occurred. Notably, we found a 3.2 times higher mortality rate (180 days observation) for patients after any femur fracture who developed TRAKI in comparison to patients with a similar femur fracture in absence of TRAKI after adjustment for potential confounders. Therefore, patients with femur fracture who develop posttraumatic TRAKI should receive special attention in peri-traumatic care to improve the long-term outcome.

The kidneys as the main actor and guarantee for a balanced fluid-electrolyte household and cross-talking organ are rather challenged after trauma: not only by altered hemodynamics and volume loss and shifts but also by accumulating tissue debris. In consequence, an abrupt decline in kidney function after trauma can occur generally defined as acute kidney injury (AKI), which can be induced by either structural damage or functional problems, or both. Clinically, two similar definitions based on enhanced concentrations of serum creatinine and reduced or absent urine output (RIFLE and AKIN) have been proposed for diagnosing and staging AKI, and subsequently, the “Kidney Disease: Improving Global Outcomes” (KDIGO) criteria for AKI were defined in 2012.^22^^,^^26^ The underlying mechanisms of trauma-related AKI (TRAKI) are driven by a complex inflammatory response resulting in alterations at the glomerular filter and tubular resorption/secretion system.^9^ Although TRAKI appears frequently after a trauma, little is known about the long-term consequences of its presence or absence.

Several other studies with much smaller cohorts but of primary data mainly focused on proximal femur fractures (hip-fractures) and reported dependent on the sampling and applied AKI criteria a prevalence between 4 and 24%: A study from Korea reported about 4% AKI development after hip fracture.^27^ One study in Turkey described about 8% AKI after hip fracture^28^ and another study in the UK even in up to 24% cases, although in the latter study, the age distribution of non-AKI versus AKI was similar to the present analysis.^19^ Whether in this context different management approaches such as geriatric co-management or timing causes such differences might have prevented the development of AKI remains speculative.^6^^,^^29^ With regard to timing, however, an interesting finding of the current study was that patients with TRAKI faced a significantly longer waiting time till surgery (even beyond 2 days) and an increased length of stay. Therefore, it is tempting to speculate that fast-track surgery might help to prevent TRAKI development. However, general complications including postoperative renal failure were not different when hip fractures were addressed within 6 versus 24 h after injury as suggested by a recent RCT.^30^ In line, we found also no differences in TRAKI development if surgery was performed on day 0 or day 1, but a significant difference if surgery was delayed by 2 or 3 or even more days, thus proposing that not fast-track but timely surgery shall help to avoid the development of TRAKI. Another factor for a significant delay in surgery of femur fractures could be reflected by an enhanced co-morbidity level and necessary preoperative optimization of the patient's condition. In this case, it is not too surprising that the more vulnerable patient will more likely develop TRAKI. This possibility needs to be excluded or addressed by a future clinical trial.

At first glance, a limitation of the study could be the significant increase of the frequency of TRAKI after femur fracture from 4.1 to 6.2% between 2014 and 2015/2016, whereas, as expected, all other basic characteristics did not differ between the observation periods. However, this was not caused by a health care management change or a possible difference in patient composition, respectively, but rather caused by the claims reporting system, which requires since 2015 an exact staging of the AKI (for adequate reimbursement). Thus, the AKI after trauma is now more precisely listed, and as a direct consequence, fewer unspecified AKI cases are listed from 2015 on. At a closer look, this might even be considered a strength of the data set which now also allows further differentiation of the posttraumatic degree of AKI e.g. in regard to the outcome which revealed an AKI stage-dependent survival (see Fig. 2). Other factors such as advanced age, female gender, degree of care need, and increased comorbidity score positively correlated with development of TRAKI. Other studies listed similar and further risk factors for development of AKI: the use of angiotensin-converting-enzyme (ACE) inhibitors or angiotensin receptor blockers, chronic kidney disease, advanced age, diabetes, hypertension, myocardial infarction, hip arthroplasty, and others.^19^ Concerning gender aspects, in contrast to our findings of female predominance in developing TRAKI, a meta-analysis on general risk factors for AKI in ICU patients considered rather male gender as a risk factor.^10^ To what extent femur fracture-specific factors might play a role here, such as the bone-kidney crosstalk e.g. in advanced osteoporosis, which certainly is influenced by gender differences, remains to be clarified. Strengths of our study were the large numbers of patients included, lack of participation and information bias, and complete mortality follow-up. Since health claims data were used in these analyses residual confounding, e.g. by physical inactivity or stress, cannot be excluded. A further limitation arose from the restricted information available from health claims data, like missing information on general functional status, clinical assessments, or laboratory values for kidney function. However, other well-established potential confounders in this research context, like need for care and comorbidity, were considered in the analyses.

To our best knowledge, this study is the first large data analysis that also addresses spatially different injury patterns of the whole femur as the underlying trigger for induction of posttraumatic AKI. Femur shaft fractures were associated with 6.1% TRAKI, followed by femur neck fractures with 5.7% and distal fractures with 4.5%, respectively. The age distribution of patients suffering from femur fracture at different locations was comparable. Taken the fact that femur shaft fractures mainly reflect high-energy trauma and are often associated with a significant blood loss up to life-threatening hemorrhagic shock, this in principle could contribute to TRAKI development. In support, experimental and clinical studies indicated blood loss-induced circulatory shock as a major driver of multiple organ failure including the kidneys.^31^^,^^32^

The present findings also point to the future. It is well established that the population is aging worldwide and the aging kidney is more susceptible to any stress.^33^ Thus, femur fractures will increase and if no therapeutic protection of the kidney such as avoidance of contrast agents, adequate treatment of shock symptoms, timely surgical intervention, avoidance of renal toxic antibiotics and drugs, etc.^9^^,^^34^^,^^35^ will be applied, it is also likely that the incidence of TRAKI will rise. To avoid AKI development, a recent study in a cardiac surgery cohort demonstrated that adherence to KDIGO-derived treatment bundles improved AKI post-operation^36^ which in principle could also be applied post-femur fracture. Furthermore, a recent machine-learning approach including novel renal damage and functional biomarkers revealed in TRAKI and burn-induced AKI an earlier prediction of AKI, which timely treated could result in an improved outcome.^37^ Future large studies could also help to follow up the consequences of TRAKI after other fracture types and other injury patterns (including polytrauma) in a long-term manner to deduce hypotheses of optimized diagnostic and treatment hypotheses for TRAKI.

## Conclusions and Implications

We found in a considerable proportion of more than 5% of the patients with femur fracture a development of TRAKI during the hospital stay. Those patients with TRAKI showed a 3.2 times higher mortality rate, even after adjustment for potential confounders. Therefore, from the clinical and preventive perspective, patients with femur fracture and compromised hemodynamics should receive special attention in peri-traumatic care such as avoidance of nephrotoxic contrast media, antibiotics and colloids, and early rebalancing of electrolyte and fluid homeostasis to prevent posttraumatic TRAKI and thus improve the long-term outcome. Those patients with femur fracture and clinical signs of TRAKI should furthermore receive continuous post-operative monitoring of kidney and related cross-talking organ functions. Future research efforts will need to focus on the influence of TRAKI on the outcome after different injury patterns such as traumatic brain injury and other diseases by further epidemiological analyses. Retranslation of such findings in association with a better understanding of the underlying immunopathophysiological mechanisms will help to define effective future therapeutic strategies.

## Funding

This work was supported by the Deutsche Forschungsgemeinschaft (DFG, German Research Foundation) – Project-ID 251293561 – SFB 1149 A01 and by the German Federal Ministry of Education and Research within the project ‘Prevention and Rehabilitation of Osteoporotic Fractures in Disadvantaged Populations 2 (PROFinD 2)’ grant no. 01EC1404A.

## Ethical approval

The ethics committee of the University of Ulm recognised that there was no necessity for ethical approval because this study comprised analysis of anonymised routine data.

## CRediT authorship contribution statement

**Gisela Büchele:** Visualization, Writing – original draft, Writing – review & editing. **Martin Rehm:** Formal analysis, Writing – review & editing. **Rebecca Halbgebauer:** Writing – original draft, Writing – review & editing. **Dietrich Rothenbacher:** Visualization, Writing – review & editing. **Markus Huber-Lang:** Visualization, Writing – original draft, Writing – review & editing.

## Declaration of Competing Interest

Gisela Büchele, Martin Rehm, Rebecca Halbgebauer, Dietrich Rothenbacher, and Markus Huber-Lang do not have any financial or personal conflicts of interest.
